# Evaluating digestion efficiency in full-scale anaerobic digesters by identifying active microbial populations through the lens of microbial activity

**DOI:** 10.1038/srep34090

**Published:** 2016-09-26

**Authors:** Ran Mei, Takashi Narihiro, Masaru K. Nobu, Kyohei Kuroda, Wen-Tso Liu

**Affiliations:** 1 Department of Civil and Environmental Engineering, University of Illinois at Urbana-Champaign, 205 North Mathews Ave, Urbana, IL 61801, USA; 2 Bioproduction Research Institute, National Institute of Advanced Industrial Science and Technology (AIST), Central 6, Higashi, Tsukuba, Ibaraki 305-8566, Japan; 3 Department of Environmental Systems Engineering, Nagaoka University of Technology, 1603-1, Kami-tomioka, Niigata 940-2188, Japan

## Abstract

Anaerobic digestion is a common technology to biologically stabilize wasted solids produced in municipal wastewater treatment. Its efficiency is usually evaluated by calculating the reduction in volatile solids, which assumes no biomass growth associated with digestion. To determine whether this assumption is valid and further evaluate digestion efficiency, this study sampled 35 digester sludge from different reactors at multiple time points together with the feed biomass in a full-scale water reclamation plant at Chicago, Illinois. The microbial communities were characterized using Illumina sequencing technology based on 16S rRNA and 16S rRNA gene (rDNA). 74 core microbial populations were identified and represented 58.7% of the entire digester community. Among them, active populations were first identified using the ratio of 16S rRNA and 16S rDNA (rRNA/rDNA) for individual populations, but this approach failed to generate consistent results. Subsequently, a recently proposed mass balance model was applied to calculate the specific growth rate (μ), and this approach successfully identified active microbial populations in digester (positive μ) that could play important roles than those with negative μ. It was further estimated that 82% of microbial populations in the feed sludge were digested in comparison with less than 50% calculated using current equations.

Municipal wastewater is treated in water reclamation plants (WRPs) by combination of physical, chemical and biological processes. Through these processes, substantial amount of wasted sludge is produced from primary and secondary clarifiers (10.9 millions tons dry solids for 27 European Union members in 2005 and estimated to exceed 13 millions in 2020^1^). In general, wasted sludge consists of a complex mixture of microorganisms, organic matters, and inorganic materials^2^. Often, wasted sludge is treated by anaerobic digestion (AD) to stabilize microbial activity and recover energy in the form of methane gas through collaborative interactions of anaerobic microorganisms, including fermenting bacteria, syntrophs and methanogens^3^.

At WRPs, the digestion efficiency is closely monitored to ensure that efficient biogas production and biohazard destruction are met, and is often calculated based on the reduction of volatile solids (VSR) after digestion^4^,^5^,^6^ with the Van Kleeck equation and the approximate mass balance equation as the two most commonly used methods^7^. Both equations assume that there is no substantial amount of biomass production during digestion. Both equations further assume that all the volatile solids (VS) in the feed are digestible, and microbes present in the wasted sludge or in the feed do not participate or proliferate during the digestion process. So far, the digestion efficiency reported varies from 20 to 70%. When the efficiency is relatively low, pretreatment technologies have been used to breakdown the cell wall structure and improve the digestion efficiency. Based on ecological understanding, low digestion efficiency also suggests another possibility that a fraction of microbes in the feed sludge can survive or participate in the digestion process and proliferate. In other words, the wasted sludge feed may not be completely digested.

To better estimate the digestion efficiency in AD, this study investigated what microbes in the feed could be digested, survived, or growing in AD by using a full-scale WRP at Chicago, Illinois as the study site. The microbial community structure was characterized using 16S rRNA (gene) based Illumina sequencing. In specific, we first investigated “core” community members that were regionally common and locally abundant in AD. Active populations in the core community were then identified using two approaches. The first approach profiled microbial activity based on 16S rRNA and 16S rDNA as a proxy for *in situ* activity and total microbial populations, respectively. The relative abundance ratio of 16S rRNA to 16S rRNA gene (rRNA/rDNA) could be further calculated to identify active microbial populations of interest. The second approach coupled relative abundance from 16S rDNA sequencing data with a mass balance model^8^, which explained microbial populations dynamics by growth, sludge input, and sludge withdraw. Using the second approach, we precisely identified populations with positive or negative specific growth rate (μ), which represented active populations that contributed to digestion or inactive populations that were digested. The findings can be further used to better estimate the digestion efficiency in AD.

## Results

### Description of water reclamation plant and anaerobic digesters

A local water reclamation plant (WRP) in Chicago, which serves 2.38 million people and has a design capacity of 1,200 million gallons per day, was selected for this study. It treats wastewater through primary and activated sludge processes (see Supplementary Fig. S1). The settled sludge from the primary and secondary clarifiers is pumped to sludge holding tanks, mixed with small amount of other sludge (partially digested sludge from the Imhoff tank and wasted sludge from another WRP). Volatile solids content of waste activated sludge (AS) accounted for 76.10% of the total feed into ADs (Supplementary Table S1), indicating it was the most significant input. Twenty-four ADs were operated and received identical blended sludge feed. In this study, digesters 1, 6, and 14 were selected, and totally 35 digester sludge samples were obtained throughout December 2014, and the last week of January 2015 (Supplementary Table S2). Table 1 summarizes the key characteristics of sampled digesters. The feed volume was 2.3 million gallons (MG) for digesters 1 and 6, and 2.8 MG for digester 14. Total solids content of the digester sludge was around 2.5%, and volatile solids fraction in the total solids content was 52%. All digesters were operated at neutral pH (7.1), mesophilic temperature (36 °C), and a retention time of 24–25 days. In sludge supernatant, nitrate and nitrite were measured but not detected. The volatile solids reduction (VSR) was 20% based on the information provided by the operator. In addition to AD samples, six AS samples were obtained from the aeration tanks located at two batteries of the WRP of a one-week interval.

### Microbial community in AD and AS

Sequences of 16S rDNA from all AD and AS samples were subsampled to 30,000 non-chimeric sequences. The rarefaction curves based on observed species and phylogenetic diversity (Supplementary Fig. S2a,b) were close to but not reaching plateau. However, the rank-abundance curve (Fig. S2c) suggested that OTUs that were more abundant than 0.0001% have been detected, suggesting major microbial populations were captured in this analysis and sufficient sequence depth was achieved. The analysis of alpha diversity (Supplementary Table S3) indicated that 51,091 and 18,413 OTUs (at 97% sequence similarity) were detected in AD and AS communities, respectively. In general, the AS community had higher diversity than the AD community based on number of OTUs per sample, Chao1 and Shannon index.

**Figure 1:**
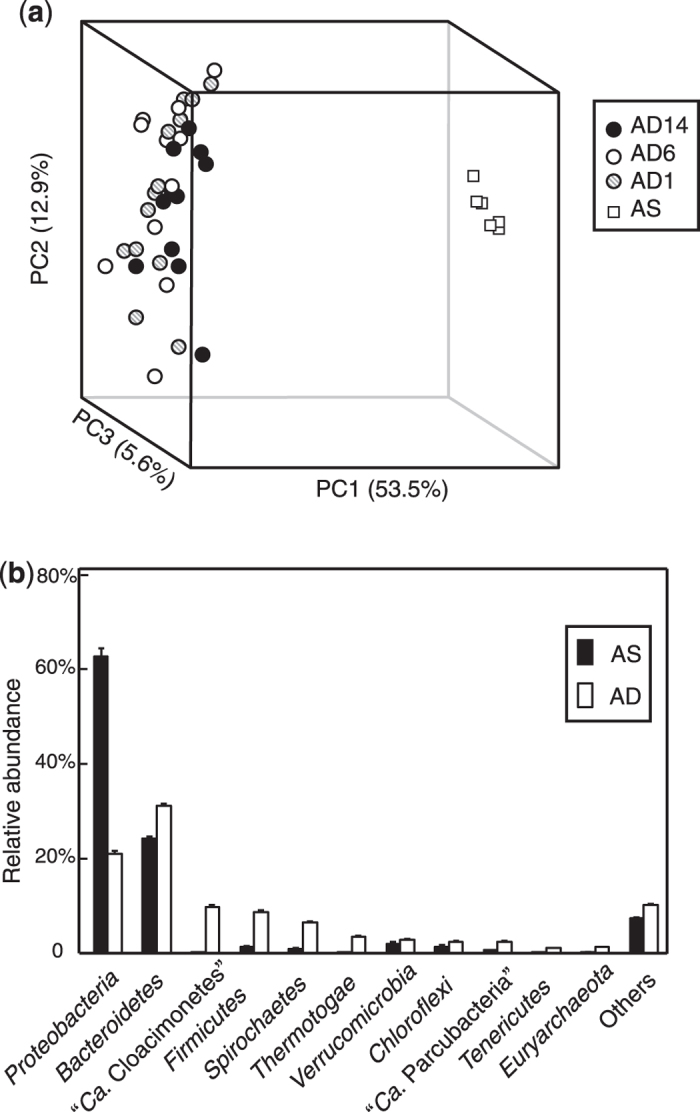
(**a**) Beta diversity of AD and AS communities based on principal coordinate analysis of weighted UniFrac distance matrix. (**b**) Community structure of AD and AS at phylum level. Phyla that were more abundant than 1% in either AD or AS were plotted. Bars indicated standard error.

The microbial community structure based on 16S rDNA of individual AD and AS samples was analyzed. Principal coordinate analysis with weighted UniFrac distance matrix indicated that there was a clear community shift from AS to AD, and that three ADs shared a similar community structure despite different sizes and different sampling days (Fig. 1a). The microbial community shift coincided with the change in the phylum-level community compositions (Fig. 1b). *Proteobacteria* was the most abundant phylum in AS (62.4%, sequence abundance) and decreased to 20.1% in AD. In contrast, *Bacteroidetes* increased from 24.1% in AS to 31.0% in AD, together with other phyla with significantly higher abundance in AD, including “*Ca*. Cloacimonetes” (formerly known as WWE1), *Firmicutes*, *Spirochaetes*, “*Ca*. Parcubacteria” (formerly OD1), *Tenericutes*, and *Euryarchaeota*.

### Core microbial populations in AD microbiome

**Figure 2:**
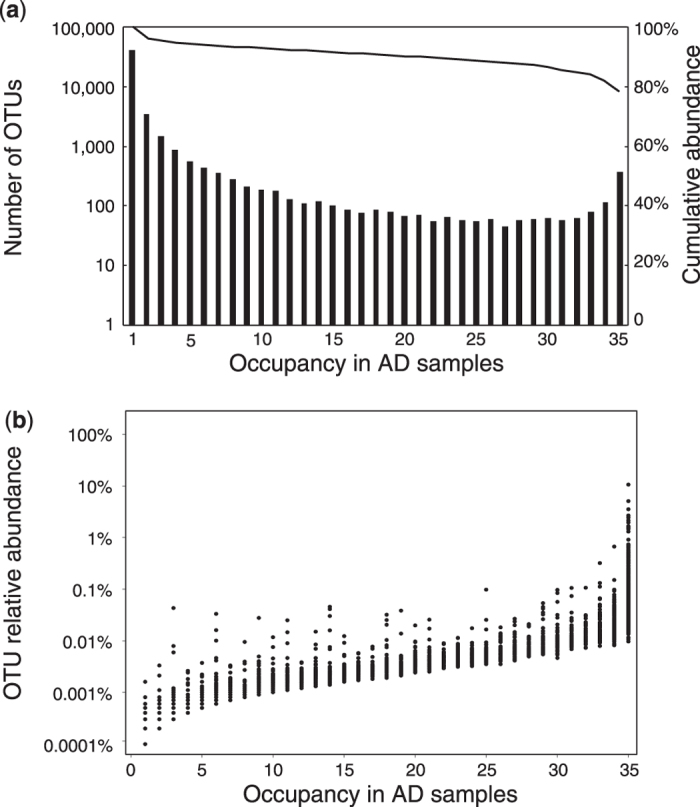
(**a**) Occupancy distribution and (**b**) occupancy-abundance correlation of AD microbial community.

To identify core microbial populations in AD, occupancy distribution and occupancy-abundance correlation, which were two prerequisites to apply the core-community model^9^, were evaluated. For a given OTU, its occupancy is defined as the number of samples where it was detected. The overall occupancy distribution (Fig. 2a) for all OTUs in AD was bimodal (Hartigan’s dip test for multimodality, p-value = 0.997): the first peak was observed at the occupancy of ≤ three samples (45,888 OTUs, 5.2% of total sequences), and the second peak at the occupancy of 35 samples (375 OTUs, 79.0% of total sequences). As the majority of sequences were represented by OTUs of the second peak, the cumulative abundance curve against occupancy did not increase drastically with the occupancy from 34 to one sample. A clear positive correlation between occupancy and abundance (Spearman’s rank correlation, p < 0.001, rho = 0.94) was observed (Fig. 2b), indicating that more frequently observed OTUs had higher abundance. For a given sample, we sorted all OTUs were in a descending order, and defined “abundant OTUs” as those that made up the top 80% of total sequences in this sample. As a consequence, 74 OTUs were assigned as “core populations” because they had occupancy of 35 samples and they were abundant in each samples. Within the 74 OTUs, 21 were closely related to known isolates (>97% sequence similarity and 100% coverage) (Supplementary Table S4): eight were obligate anaerobes, including *Methanosaeta, Smithella*, and *Syntrophorhabdus*, and 13 OTUs were either aerobes or facultative anaerobes, including *Zoogloea*, *Thauera* and *Dechloromonas*. The remaining 53 OTUs were not closely related to sequences of known isolates (Supplementary Table S5).

### rRNA/rDNA ratio failed to characterize microbial activity

**Figure 3:**
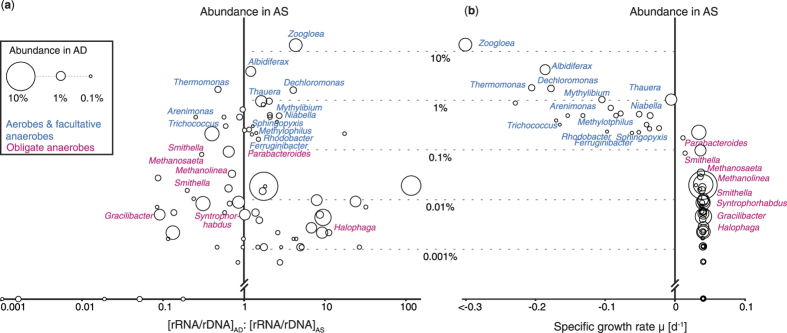
Identification of active populations within core AD community (74 OTUs) by (**a**) comparing rRNA/rDNA ratio in AD and AS (**b**) calculating specific growth rate μ using mass balance model. OTUs with closely related isolates were labeled, and colored based on the physiology of related isolates. Bubble size represented abundance in AD. y axis indicated abundance in AS. x axis in (**a**) indicated the results of rRNA/rDNA ratio in AD divided by the ratio in AS. x axis in (**b**) indicated the specific growth rate μ calculated by mass balance model.

For each OTU in the core populations, the ratio of the relative abundance of 16S rRNA to the abundance of 16S rRNA gene (rRNA/rDNA) was calculated in both AS and AD. Thus, the value of [rRNA/rDNA]_AD_:[rRNA/rDNA]_AS_ for a given OTU could be used to determine whether this OTU was more active in AD or in AS (Fig. 3a). OTUs with high abundance in AS (shown as large value on Y axis in Fig. 3a) were observed to have higher ratio in AD than in AS (i.e., [rRNA/rDNA]_AD_:[rRNA/rDNA]_AS_ > 1), suggesting that they were more active in AD than in AS. This included eight OTUs that were closely related to aerobes or facultative anaerobes, such as *Zoogloea*, *Thauera*, and *Dechloromonas*. In contrast, we observed that OTUs with high abundance in AD had lower ratio in AD than in AS ([rRNA/rDNA]_AD_:[rRNA/rDNA]_AS_ < 1), suggesting that they were less active in AD than in AS. Some of these OTUs were obligate anaerobes like *Methanosaeta* and *Smithella*. Clearly the use of the rRNA/rDNA ratio to identify active populations in AD may not be adequate.

### Microbial activity calculated by mass balance model

As an alternative approach to identify active populations in AD, a mass balance model was introduced to determine the specific growth rate (μ) of individual OTUs. Among the 74 core OTUs identified (Fig. 3b), 22 exhibited negative μ, accounting for 10% of total 16S rDNA sequences in AD. Thirteen of the 22 OTUs were closely related to known aerobes and facultative anaerobes. OTU17158, which is related to *Zoogloea*, had the lowest μ (<−0.3 d^−1^), followed by *Thermomonas*, *Albidiferax*, and *Dechloromonas*-related OTUs. The remaining 52 OTUs with positive μ accounted for 48.8% of total sequences in AD. They were more abundant in AD than in AS, including eight OTUs that were closely related to known obligate anaerobes. The values of μ varied from 0.011 to 0.041 d^−1^. 18 of the 52 OTUs reached a μ of 0.041 d^−1^, which was the maximal value of μ calculated by this model. 31 OTUs had a value of μ between 0.035 and 0.04 d^−1^, and only three OTUs had a value of μ between 0.01 to 0.03 d^−1^.

**Figure 4:**
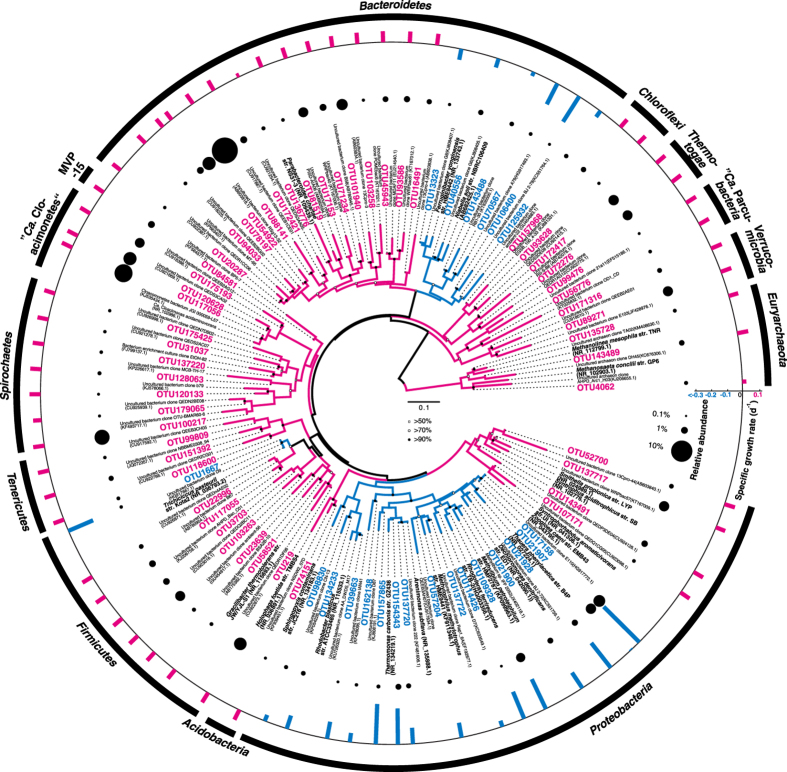
Phylogenetic distribution of 74 core OTUs with their corresponding abundance and specific growth rate μ in AD. Phylogenetic tree in the polar format was constructed using the methods of neighbor joining and parsimony. Reference sequences from environmental samples were in grey, and those from isolated cultures were in black. OTUs with positive μ were colored in purple, and those with negative μ were in blue. Branch in purple or blue indicated this clade likely contained microbes with positive or negative μ. Branch in black indicated this clade likely contained both. Bubbles in the middle layer represented relative abundance in AD of corresponding OTUs. Bars in the outer layer represented specific growth rate μ of corresponding OTUs.

Phylogenetic affiliation of the 74 core OTUs revealed a clear pattern of clustering in corresponding to μ (Fig. 4). It was observed that those 22 OTUs with negative μ were associated with two clades: one with *Beta*-, *Gamma*-, and *Alphaproteobacteria*, and the other with *Chitinophagaceae, Saprospiraceae*, *Cryomorphaceae*, and *Cytophagaceae* in the aerobic *Bacteroidetes* clades. Only OTU1667 with negative μ was not in the above two clades, and was related to *Trichococcus* with isolates known as facultative anaerobes.

None of the 52 OTUs with positive μ were associated with the two clades described above. They were affiliated with ten different phyla. *Euryarchaeota*, *Proteobacteria*, *Bacteroidetes*, “*Ca*. Cloacimonetes”, *Firmicutes*, and *Spirochaetes* contained more than three OTUs in each phylum. Three OTUs from *Euryarchaeota* were all methanogens: the most abundant one (0.43%) was related to the recently proposed class “*Ca*. Methanofastidiosa” (formerly WSA2 or Arc1), and the remaining two to *Methanolinea* and *Methanosaeta*. Four OTUs (1.5%, most abundant) in *Proteobacteria* were all related to syntrophic bacteria from *Deltaproteobacteria*, including *Smithella* and *Syntrophorhabdus*. Within *Bacteroidetes*, 16 OTUs were identified with positive μ, including the most abundant OTU (9.8%) in the AD community, and were all from the order *Bacteroidales*. They formed a distinct cluster different from the other OTUs from another distinct cluster with the phylum that had negative μ. “*Ca*. Cloacimonetes” contained three OTUs, and the most abundant one (4.7%) was closely related to “*Ca*. Cloacamonas acidaminovorans”. *Firmicutes* and *Spirochaetes* both contained seven OTUs with the most abundant one at 2.3% and 3.3%, respectively. *Tenericutes*, *Acidobacteria*, MVP-15, *Thermotogae*, *Chloroflexi*, “*Ca*. Parcubacteria”, and *Verrucomicrobia* only contained three or less OTUs with positive μ.

We confirmed the results of growth rate by measuring the absolute abundance (16S rRNA gene copy number) of specific taxa using quantitative PCR (Supplementary Fig. S3). Major OTUs in *Syntrophaceae* and *Archaea* exclusively had positive μ, and major OTUs in *Betaproteobacteria* exclusively had negative μ. Thus these three taxa were chosen to compare absolute abundance in AS and AD. The abundances of *Syntrophaceae* and *Archaea* were higher in AD than in AS, suggesting they were growing during digestion. In contrast, the abundance of *Betaproteobacteria* were much lower in AD than in AS, suggesting it was digested during digestion.

### Digestion efficiency of wasted activated sludge

**Figure 5:**
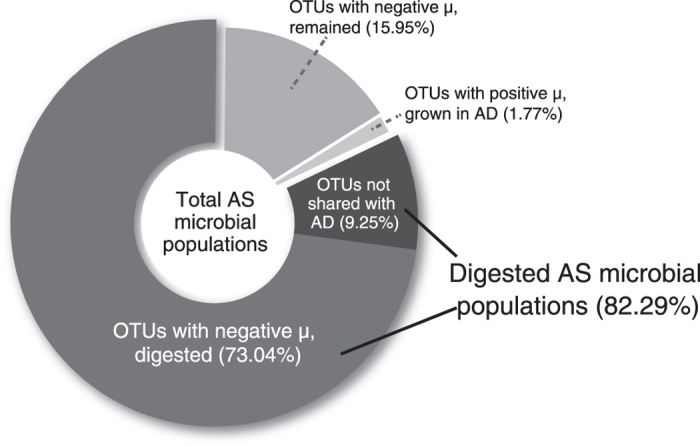
Determination of digestion efficiency based on microbial activity.

We applied the information of μ to calculate to what extent microbial populations in AS were digested (Fig. 5). Among all the 18,413 OTUs detected in AS, 13,749 OTUs (9.25% of total sequences in AS) were not detected in AD, suggesting that they were digested or their abundances were too low to be detected. Among the 4,664 OTUs that were shared with AD, 456 (1.77%) had positive μ. The remaining 4,208 OTUs had negative μ, and the fraction that was digested in AD accounted for 73.04% of sequences of AS community (see Methods section for detailed calculation). Based on these calculations, we estimated that 82.29% (9.25% + 73.04) of total populations present in AS community were digested. The digestion efficiency was also calculated based on conventional equations that consider VSR before and after digestion. Based on Van Kleeck equation (equation 4) and approximate mass balance equation (equation 5), the digestion efficiency was 42.02% and 34.99%, respectively. These numbers were a lot lower than that obtained by the mass balance model that assumed a certain fraction of the biomass in the feed could grow in AD. The discrepancy between values calculated by mass balance model and conventional equations was contributed by new-growing biomass (populations with positive μ), which accounted for 75% sequences in the digested sludge.

## Discussion

In a microbial ecosystem, the occurrence and abundance of species inhabiting is often associated to identify the core microbial populations. Using the widely applied “core-satellite” model as an example, if a species is detected in all samples sites (i.e., high occupancy) with high abundance, it is classified as a core member that can play important ecological functions^9^. This model works well in ecosystems where migration of source organisms is minimal (e.g., natural forest where plants are not able to move^9^, microbiome in drinking water distribution system where biofilm is locally developed^10^, bacterial community in cystic fibrosis lung where there is few microbes coming in with air^11^). However, an anaerobic digester is not an isolated system, as it receives massive influx of microorganisms from solids discharged from activated sludge processes. When the digestion process is not complete, a significant portion of microbes in the feed biomass can still be detected in AD and mistakenly identified as “core populations” especially using DNA-based molecular technologies. To address this drawback, this study considered the microbial activities of individual populations present in AD.

To evaluate microbial activity, the method of 16S rRNA/rDNA, which was used in many studies especially in marine ecosystems^12^,^13^,^14^, was applied first. We compared the ratio of a given OTU in the AS fed and its own ratio in AD, in order to determine whether this OTU was more active in AS or in AD. We found that aerobic and facultative anaerobic taxa had higher rRNA/rDNA ratio in AD than in AS, and obligate anaerobic taxa had lower ratio in AD than in AS. However, in AD, which was a methanogenic environment, aerobes and facultative anaerobes should have low metabolic activities due to limited amount of external electron acceptors like oxygen or nitrate, and obligate anaerobes should have higher activities. It is clear that this known microbial physiology was inconsistent with results generated using the rRNA/rDNA ratio.

**Figure 6:**
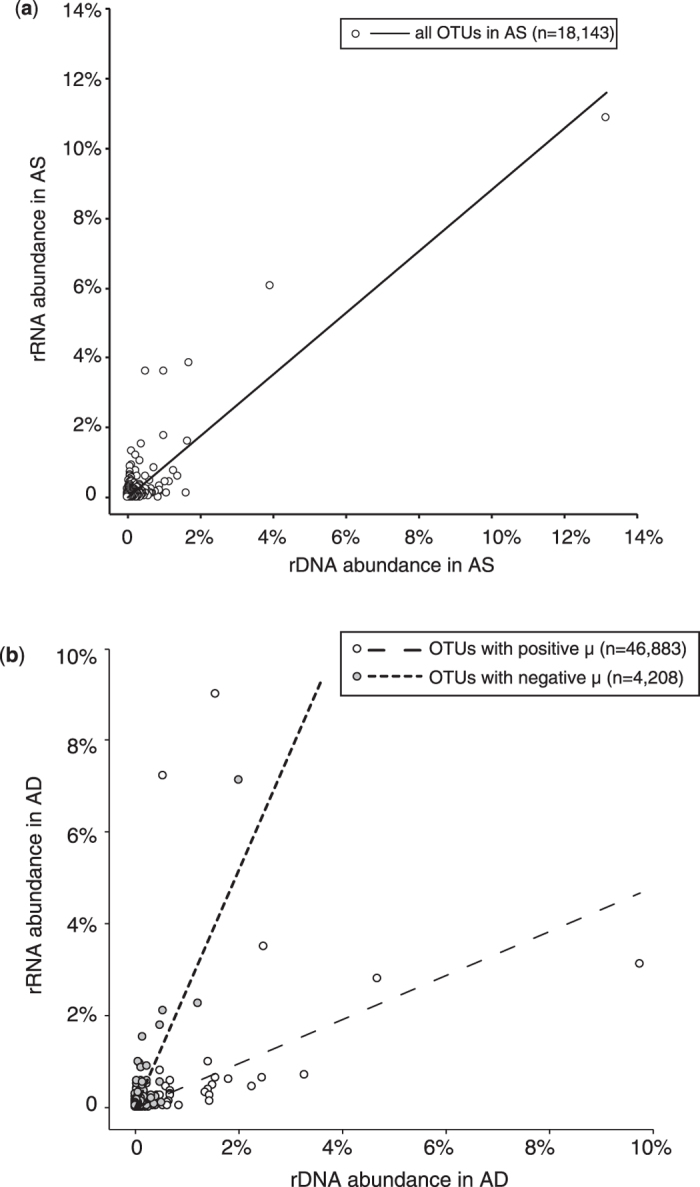
Correlation of relative abundance of 16S rRNA and 16S rDNA in (**a**) AS and (**b**) AD.

We reasoned that the inability to use 16S rRNA/rDNA ratio to identify active populations was due to the improper use of rRNA/rDNA ratio calculated from relative abundance data. For a given microorganism, absolute abundance of 16S rRNA is used to represent its total activity and the absolute abundance of 16S rDNA is used to represent its population size. Thus the ratio of rRNA/rDNA is an normalized indicator of the unit activity of this microorganism^15^. When relative abundance is used instead of absolute abundance to calculate the ratio, it is critical that the overall correlation of rRNA relative abundance to rDNA relative abundance in AS and AD are the same (see Supplementary Information). However, our dataset showed that the correlation of rRNA relative abundance with rDNA relative abundance in AS was different with the correlation in AD (Fig. 6). The rRNA and rDNA relative abundance were highly correlated in AS (Pearson’s product-moment correlation test, cor = 0.873, n = 18,413) but not in AD (cor = 0.496, n = 51,091). In fact, there were two distinct correlations in AD that differed significantly in slope (ANCOVA, p < 0.001): one was constituted with populations with positive μ (cor = 0.820, n = 46,883), and the other with populations with negative μ (cor = 0.869, n = 4,208). This was due to the fact that in AD microbiome, a large fraction of populations consisted of “alien” microorganisms that migrated from AS. These microbes of negative μ in AD differed decisively with native AD microbes of positive μ, in term of physiology. Such difference has been reported to lead to different rRNA/rDNA ratio^16^, which was observed here as two distinct rRNA/rDNA correlations coexisting in AD. This heterogeneity determined the rRNA/rDNA ratio approach relying on relative abundance was not adequate here.

As an alternative approach to identify active populations, the mass balance model and DNA-based relative abundance were combined and used. Intriguingly, this approach successfully identified organisms that were likely inactive in AD, despite they were classified as core populations. For core OTUs with negative μ within *Proteobacteria* (except for *Deltaproteobacteria*), *Zoogloea*, *Trichococcus* and *Dechloromonas* were observed as the top abundant genera in activated sludge from sewage treatment plants in Asia, North America, and Europe^8^,^17^. Their occurrence in AD is the result of AS residue but not anaerobic metabolism. Similarly, for OTUs with negative μ within the phylum *Bacteroidetes*, they were related to *Chitinophagaceae, Cryomorphaceae*, *Cytophagaceae*, and *Saprospiraceae*, which generally grow under aerobic conditions^18^.

We further examined core OTUs with positive μ. Some were closely related to obligate anaerobes, including methanogens (*Methanosaeta* and *Methanolinea*) and syntrophs (*Smithella* and *Syntrophorhabdus*). For those OTUs without closely related isolates, they were associated with taxa that were capable of anaerobic metabolism and frequently detected in anaerobic bioreactors^19^. For example, “*Ca*. Methanofastidiosa” (WSA2) was recently proposed to perform methyl-reducing for methane production^20^, and “*Ca*. Cloacimonetes” (WWE1) was suggested to perform amino acids fermentation as well as syntrophic propionate oxidation^21^. *Bacteroidales*, *Firmicutes* clade SHA-98, *Spirochaetes* clade SA-8, and *Chloroflexi* subphylum I were all considered to perform fermentation of various organic matters^22^,^23^,^24^,^25^,^26^. This information supported the mass balance model that populations with positive μ were very likely to contribute to anaerobic digestion.

At present, digestion efficiency based on VSR, calculated by Van Kleeck equation or approximate mass balance equation, could likely underestimate digestion efficiency (35–42% volatile solids reduction), because they assumed digestion as a process where only degradation (no growth) with feed biomass can occur. Our study indicated that the major fraction of microbial populations in digester could grow actively, as represented by OTUs with positive μ. Being able to discriminate active and inactive populations in AD, the growth model used in this study estimated that 82% of microbial populations in AS were digested, which was greater than the results from the conventional equations. The remaining undigested microbial populations of the feed biomass suggest the need for specific pretreatment technologies. For example, *Zoogloea* is identified as the major undigested AS population. It can accumulate polyhydroxyalkanoates in cell and secrete complex extracellular polysaccharides^27^. Thus, it will require special pretreatment to decompose the high-molecular-weight polymers and improve digestion efficiency.

## Methods

### Sample preparation and sequencing

Samples for AD were collected from the recycle line. The port was open for one minute to ensure that sludge taken could represent the sludge of the entire digester with proper temperature. Then 50 ml of sludge was collected. Samples for AS were directly collected from the aeration tanks. They were stored at −20 °C before further analyses. Nitrate and nitrite were measured with a ICS-2100 IC system (ThermoFisher Scientific). Two milliliters of well-mixed liquid were used for DNA or RNA extraction. Genomic DNA for a given sample was extracted using the FastDNA SPIN Kit for Soil (MP Biomedicals, Carlsbad, CA, USA) according to the manufacturer’s instructions, and quantified using a Nanodrop 2000c spectrophotometer. For PCR amplification, 60 ng of genomic DNA was added into a total reaction volume of 25 μL as template. With a dual-indexing approach^28^, a universal primer set 515F (5′-GTGCCAGCMGCCGCGGTAA-3′)/909R(5′-CCCCGYCAATTCMTTTRAGT-3′) targeting the V4-V5 region of 16S rRNA gene was used for amplifying both bacterial and archaeal 16S rRNA gene^29^. PCR was performed with the thermal cycling protocol consisting of initial denaturation (94 °C, 3 min), 25 cycles of denaturation (94 °C, 30 s), annealing (55 °C, 45 s) and extension (72 °C, 1 min), and a final extension (72 °C, 10 min). The PCR amplicons were purified using the Wizard SV Gel and PCR Clean-Up system (Promega, Fichburg, WI, USA) and quantified by Qubit 2.0 Fluorometer.

RNA was extracted and purified using acid-phenol/chloroform/isoamyl alcohol (125:24:1) and chloroform, and precipitated by ethanol at −80 °C^30^. DNase treatments were performed with both RNase-free DNase Set (Qiagen, Valencia, CA, USA) and Turbo DNA-free (Ambion, Austin, TX, USA). DNA-targeting PCR was carried out to detect genomic DNA contamination in the purified RNA. Reverse transcription and 16S rRNA gene amplification were performed simultaneously with the primer set described above and the Superscript III One Step RT-PCR with Platinum *Taq* (Invitrogen, Carlsbad, CA, USA). The thermal cycling protocol consisted of cDNA synthesis (55 °C, 30 min), initial denaturation (94 °C, 2 min), 30 cycles of denaturation (94 °C, 15 s), annealing (55 °C, 30 s) and extension (68 °C, 1 min), and a final extension (68 °C, 5 min). The amplicons were purified and quantified as described above. PCR/RT-PCR products from each sample were mixed to get a pooled amplicon library with a final concentration of approximately 10 ng/μL. Sequencing was performed on Illumina Miseq Bulk 2 × 300 nt paired-end system at the Roy J. Carver Biotechnology Center at the University of Illinois at Urbana-Champaign.

### Operational taxonomic unit analysis

Raw sequences after sequencing were assembled, screened, and trimmed using Mothur 1.33.3^31^ with maximum sequence length of 400 bp and a quality score of 20. The output data were analyzed using QIIME 1.9.1^32^ for OTU picking with the *de novo* strategy. UCLUST^33^ was used to group sequences into OTUs based on the similarity against Greengenes database with 97% identity cut-off. For each OTU, the most abundant sequence was selected as the representative sequence, and was further aligned using PyNAST^34^. Chimeric sequences were removed using ChimeraSlayer^35^. The taxonomy was assigned by both UCLUST and BLAST^36^ with a maximum e-value of 0.001. Alpha diversity indices and principal coordinate analysis (PCoA) of UniFrac distance matrix was performed using QIIME. Phylogenetic tree of major OTUs was constructed using the methods of neighbor joining and parsimony provided in ARB program^37^. Reference sequences in the tree were downloaded from NCBI nucleotide collection database and all sequences were longer than 1,300 bp. Relative abundance was calculated based on OTU table generated by QIIME after subsampling to even depth of 30000 sequences per sample for 16S rRNA gene and 10000 per sample for 16S rRNA. Statistical tests (e.g. Hartigan’s dip test for multimodality, Spearman’s rank correlation, Pearson’s product-moment correlation, Welch test, analysis of covariance (ANCOVA), Wilcoxon test) were performed using R^38^. OTUs with closely related isolates were confirmed using BLAST and their physiology was obtained from literature (supplementary information). The sequence data obtained in this study were deposited at DDBJ under accession no. DRA004569.

### Calculation of specific growth rate (μ)

The mass balance calculation was modified from a recent study^8^. We defined anaerobic digester as control volume for mass balance calculation and used volatile suspended solids concentration (VSS) to approximate cell concentration^4^. For a microbial population (x) or OTU, the description equation can be stated as:


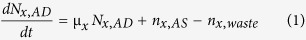


where, *N*_*x,AD*_ is absolute number of microorganism x in AD [−]; μ_*x*_ is specific growth rate constant for microorganism x [d^−1^]; 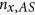 is number of microorganism x in activated sludge entering AD per day [d^−1^]; and 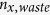 is number of microorganism x in wasted sludge leaving AD per day [d^−1^]. Specific growth rate of individual OTUs were calculated for three digesters separately and averaged for further analyses. Detailed information for calculation is available in supplementary information.

### Calculation of digestion efficiency

This study considered digested AS populations as: (1) OTUs that were detected in AS but not in AD and (2) OTUs that were detected in AD but had negative growth. The fraction of part (1) in AS community was calculated as


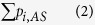


where *p*_*i,A,S*_ is the DNA-based relative abundance of OTU i in AS.

For an OTU j in part (2), its biomass in AS could be expressed as *Q*_*AS*_*TS*_*AS*_*VS*_*AS*_*p*_*j*_,_*AS*_. Its remaining biomass after digestion is *Q*_*AD*_*TS*_*AD*_*VS*_*AD*_*p*_*j*_,_*AD*_. Here Q_AS_ is the feed sludge flow rate, and Q_AD_ is the output AD sludge flow rate. TS stands for total solids content, VSS as volatile solids, and p as DAN-based relative abundance. Thus the digested biomass of OTU j was *Q*_*AS*_*TS*_*AS*_*VS*_*AS*_*p*_*j*_,_*AS*_ − *Q*_*AD*_*TS*_*AD*_*VS*_*AD*_*p*_*j*_,_*AD*_. The total amount of digested biomass of this part was Σ(*Q*_*AS*_*TS*_*AS*_*VS*_*AS*_*p*_*j*_,_*AS*_ − *Q*_*AD*_*TS*_*AD*_*VS*_*AD*_*p*_*j*_,_*A*_) and the fraction in AS community was calculated as:


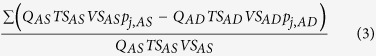


Thus, total digested AS population could be defined as the sum of part (1) and (2).

In addition, digestion efficiency based on volatile solid reduction was calculated by Van Kleeck equation 
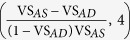
 and approximate mass balance equation 
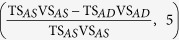
39.

### Quantitative PCR (qPCR)

qPCR assays were performed targeting the 16S rRNA genes of total *Bacteria*, family *Syntrophaceae*, domain *Archaea*, and class *Betaproteobacteria*. Serial 1:10 dilutions of plasmid containing cloned 16S rRNA genes (for total *Bacteria* and *Syntrophaceae*) or synthesized DNA fragments (for *Archaea* and *Betaproteobacteria*) aqueous solution were used as external standards. Reactions were carried out in a StepOnePlus Real-Time PCR System (Applied Biosystems) using the Power SYBR Green PCR Mater Mix (Applied Biosystems). The reaction volume was 20 μl. Primer sets and reaction condition were shown in Supplementary Table S6. One cycle of initial denaturing at 95 °C for 15 min was performed first, followed by 40 cycles of amplification. Analysis of melt curves (in steps of 0.5 °C for 5 s, with temperatures ranging from 60 to 95 °C) was performed after amplification. Reaction of each sample was performed in duplicates.

## Additional Information

**How to cite this article**: Mei, R. *et al*. Evaluating digestion efficiency in full-scale anaerobic digesters by identifying active microbial populations through the lens of microbial activity. *Sci. Rep*. **6**, 34090; doi: 10.1038/srep34090 (2016).

## Supplementary Material

Supplementary Information

## Figures and Tables

**Table 1:** Characteristics of the three sampled digesters in Stickney WRP.

AD	Volume (million gallon)	Feed (million gallon per day)	Total solids (%)	Volatile solids (%)	Volatile solids reduction (%)	pH	Temperature (°C)	Sludge retention time (day)
1	2.3	0.106 ± 0.003	2.57 ± 0.11	51.5 ± 0.6	23.9 ± 2.3	7.20 ± 0.07	35.8 ± 0.2	24.2 ± 0.8
6	2.3	0.106 ± 0.003	2.25 ± 0.04	53.5 ± 0.3	21.7 ± 1.8	7.14 ± 0.04	36.4 ± 0.1	24.2 ± 0.8
14	2.8	0.120 ± 0.002	2.65 ± 0.06	52.9 ± 0.5	19.8 ± 2.0	7.10 ± 0.06	36.2 ± 0.2	25.2 ± 0.4
