# Supplementary material for: Evaluating digestion efficiency in full-scale anaerobic digesters by identifying active microbial populations through the lens of microbial activity
Source: Sci Rep. 2016 Sep 26;6:34090. doi: 10.1038/srep34090 (PMC5036182; doi:10.1038/srep34090)
Supplement: Supplementary Information [file srep34090-s1.pdf]

Evaluating digestion efficiency in full-scale anaerobic digesters by identifying active microbial populations through the lens of microbial activity

Ran Mei[1], Takashi Narihiro[1,2], Masaru K. Nobu[1], Kyohei Kuroda[1,3], Wen-Tso Liu[1*]

[1]Department of Civil and Environmental Engineering, University of Illinois at Urbana-Champaign, 205 North Mathews Ave, Urbana, IL 61801, USA

[2]Bioproduction Research Institute, National Institute of Advanced Industrial Science and Technology (AIST), Central 6, Higashi, Tsukuba, Ibaraki 305-8566, Japan

[3]Department of Environmental Systems Engineering, Nagaoka University of Technology, 1603-1, Kami-tomioka, Niigata 940-2188, Japan

* E-mail address: wtliu@illinois.edu (Wen-Tso Liu).

Calculation of net growth rate

With the mass balance model

$$\frac{dN_{x,AD}}{dt} = \mu_x N_{x,AD} + n_{x,AS} - n_{x,waste} \qquad \text{(S1)}$$

where

$N_{x,AD}$	absolute number of microorganism x in AD [-]
μ_x	specific growth rate for microorganism x [d^{-1}]
$n_{x,AS}$	number of microorganism x in activated sludge entering AD per day [d^{-1}]
$n_{x,waste}$	number of microorganism x in wasted sludge leaving AD per day [d^{-1}]

When the digester is running at steady state, there is no net change of cell number of x in AD. Thus $\frac{dN_{x,AD}}{dt} = 0$, and (equation S1) can be arranged as

$$\mu_x = \frac{n_{x,waste} - n_{x,AS}}{N_{x,AD}} \qquad \text{(S2)}$$

Cell number of x in wasted sludge, AS and AD can be calculated by it relative abundance $p_{x,waste}$, $p_{x,AS}$ and $p_{x,AD}$. Equation S2 thus can be expanded as:

$$\mu_x = \frac{p_{x,waste} n_{waste} - p_{x,AS} n_{AS}}{p_{x,AD} N_{AD}} = \frac{p_{x,waste}}{p_{x,AD}} \frac{n_{waste}}{N_{AD}} - \frac{p_{x,AS}}{p_{x,AD}} \frac{n_{AS}}{N_{AD}} \qquad \text{(S3)}$$

$p_{x,waste}$ can be treated equal to $p_{x,AD}$. $p_{x,AS}$ and $p_{x,AD}$ can be obtained from OTU table.

$\frac{n_{waste}}{N_{AD}} = \frac{1}{SRT}$ where SRT is sludge retention time.

$\frac{n_{AS}}{N_{AD}} = \frac{C_{AS} Q_{AS}}{C_{AD} V_{AD}} = \frac{TS_{AS} VS_{AS}}{TS_{AD} VS_{AD}} \frac{Q_{AS}}{V_{AD}}$, Q_{AS} is the volumetric feeding rate of activated sludge. V_{AD} is the volume of anaerobic digester. C_{AS} is the total cell concentration in AS (L^{-1}), and C_{AD} is the total cell concentration in AD (L^{-1}). Here concentration of volatile solids (g/L) was used as proximity to total cell concentration (L^{-1}), and the unit in numerator and denominator cancelled out. Concentration of volatile solids (g/L) was calculated as the product of TS (total solids, g/L) and VS (volatile fraction in total solids, %). Total cell concentration of AS and AD was alternatively calculated by qPCR targeting total bacterial 16S rDNA. Resulted copy number was used to substitute "concentration of volatile solids (g/L)" in equation to calculated a new value of specific growth rate (μ'). For the 74 core OTUs, we performed paired Wilcoxon test to compare each pair of μ and μ'. The result (p-value=0.74) indicated the results based on qPCR are statistically same to the results based on volatile solids concentration.

Now we obtained the equation to calculate specific growth rate of one microorganism x as:

$$\mu_x = \frac{1}{SRT} - \frac{p_{x,AS}}{p_{x,AD}} \frac{TS_{AS}VS_{AS}}{TS_{AD}VS_{AD}} \frac{Q_{AS}}{V_{AD}} \quad \text{(S4)}$$

The final growth rate of microorganism x is averaged using values calculated from three digesters since all the digesters received identical feeding sludge and were operated under same condition, and the community structures of three digesters were very similar based on beta-diversity (Fig. 1).

Evaluation of rRNA/rDNA ratio approach

System	Index	OTU X_i with μ<0		OTU Y_j with μ>0	
		Absolute abundance	Relative abundance	Absolute abundance	Relative abundance
Activated sludge	rRNA	asR_{X_i}	$\frac{asR_{X_i}}{asR_X + asR_Y}$	asR_{Y_j}	$\frac{asR_{Y_j}}{asR_X + asR_Y}$
	rDNA	asD_{X_i}	$\frac{asD_{X_i}}{asD_X + asD_Y}$	asD_{Y_j}	$\frac{asD_{Y_j}}{asD_X + asD_Y}$
	rRNA/rDNA ratio	$\frac{asR_{X_i}}{asD_{X_i}}$	$\frac{asR_{X_i}}{asD_{X_i}} \cdot \frac{asD_X + asD_Y}{asR_X + asR_Y}$	$\frac{asR_{Y_j}}{asD_{Y_j}}$	$\frac{asR_{Y_j}}{asD_{Y_j}} \cdot \frac{asD_X + asD_Y}{asR_X + asR_Y}$
Anaerobic digester	rRNA	adR_{X_i}	$\frac{adR_{X_i}}{adR_X + adR_Y}$	adR_{Y_j}	$\frac{adR_{Y_j}}{adR_X + adR_Y}$
	rDNA	adD_{X_i}	$\frac{adD_{X_i}}{adD_X + adD_Y}$	adD_{Y_j}	$\frac{adD_{Y_j}}{adD_X + adD_Y}$
	rRNA/rDNA ratio	$\frac{adR_{X_i}}{adD_{X_i}}$	$\frac{adR_{X_i}}{adD_{X_i}} \cdot \frac{adD_X + adD_Y}{adR_X + adR_Y}$	$\frac{adR_{Y_j}}{adD_{Y_j}}$	$\frac{adR_{Y_j}}{adD_{Y_j}} \cdot \frac{adD_X + adD_Y}{adR_X + adR_Y}$
$\frac{ratio_{as}}{ratio_{ad}}$		$\frac{asR_{X_i}}{asD_{X_i}} \cdot \frac{ad\square_{X_i}}{adR_{X_i}}$	$(\frac{asR_{X_i}}{asD_{X_i}} \cdot \frac{adD_{X_i}}{adR_{X_i}}) \cdot (\frac{asD_X + asD_Y}{asR_X + asR_Y} \cdot \frac{adR_X + adR_Y}{adD_X + adD_Y})$	$\frac{asR_{Y_j}}{asD_{Y_j}} \cdot \frac{adD_{Y_j}}{adR_{Y_j}}$	$(\frac{asR_{Y_j}}{asD_{Y_j}} \cdot \frac{adD_{Y_j}}{adR_{Y_j}}) \cdot (\frac{asD_X + asD_Y}{asR_X + asR_Y} \cdot \frac{adR_X + adR_Y}{adD_X + adD_Y})$

1. Based on growth rate calculation, OTUs with negative growth in AD are denoted as X_1, X_2, ... X_i ... OTUs with positive growth are denoted as Y_1, Y_2, ... Y_j ...
2. RNA and DNA abundance in AS and AD are prefixed with *as* and *ad*, respectively
3. Total RNA in AS was expressed as $asR = asR_X + asR_Y = \sum_{i=1} asR_{X_i} + \sum_{j=1} asR_{Y_j}$, total DNA in AS was expressed as $asD = asD_X + asD_Y$, total RNA in AD was expressed as $adR = adR_X + adR_Y$, total DNA in AD was expressed as $adD = adD_X + adD_Y$

For OTU X_i, the rRNA/rDNA ratio of absolute abundance should be used to compare the actual activity in AS and AD.

$$k_{X_i} = \left[\frac{ratio_{as}}{ratio_{ad}}\right]_{absolute} = \frac{asR_{X_i}}{asD_{X_i}} \cdot \frac{adD_{X_i}}{adR_{X_i}}$$

However, we are using the ratio from relative abundance, where the comparison becomes

$$k_{X_i}' = \left[\frac{ratio_{as}}{ratio_{ad}}\right]_{relative} = \left(\frac{asR_{X_i}}{asD_{X_i}} \cdot \frac{adD_{X_i}}{adR_{X_i}}\right) \cdot \left(\frac{asD_X + asD_Y}{asR_X + asR_Y} \cdot \frac{adR_X + adR_Y}{adD_X + adD_Y}\right) = k_{X_i} \cdot C$$

where $C = \frac{asD_X+asD_Y}{asR_X+asR_Y} \cdot \frac{adR_X+adR_Y}{adD_X+adD_Y} = \frac{asD}{asR} \cdot \frac{adR}{adD}$, which is universally constant for all OTUs.

If we want to use $k_{X_i}' = k_{X_i} \cdot C$ as equivalent to k_{X_i}, it must be true that

for any k_{X_i}

when $0 < k_{X_i} < 1$, $k_{X_i} \cdot C \ < 1$;

and when $k_{X_i} > 1$, $k_{X_i} \cdot C > 1$.

So C=1

We mathematically prove C=1 as follows:

According to infimum and supremum principle,

For $\forall k > 1, C \geq \inf\{\frac{1}{k} | k > 1\} = 1$

$\forall 0 < k < 1, C \leq \sup\{\frac{1}{k} | 0 < k < 1\} = 1$

$\therefore C \geq 1, and\ C \leq 1$

$\therefore C = 1$

Thus, $\frac{asD}{asR} \cdot \frac{adR}{adD}$ should equal to 1 if we want to use the relative abundance ratio k_{X_i}' to substitute absolute abundance ratio k_{X_i}.

If the correlation of RNA and DNA in AS is $cor_{as} = \frac{asR}{asD}$, and the correlation in AD is $cor_{ad} = \frac{adR}{adD}$, then cor_{as} should equal cor_{ad}.

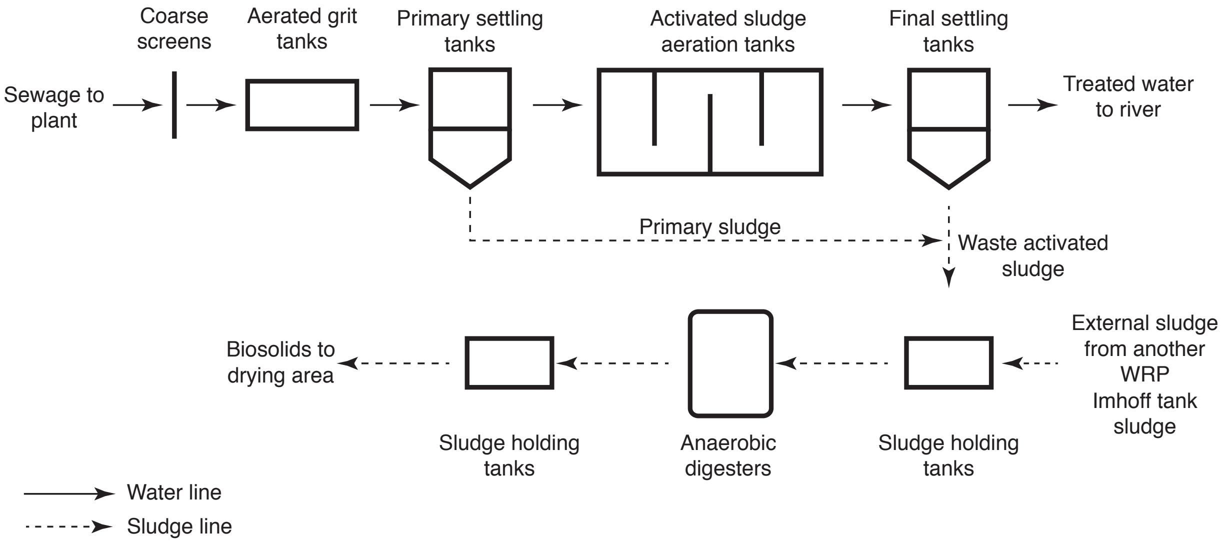

Supplementary Figure S1. Schematic configuration of Stickney Water Reclamation Plant.

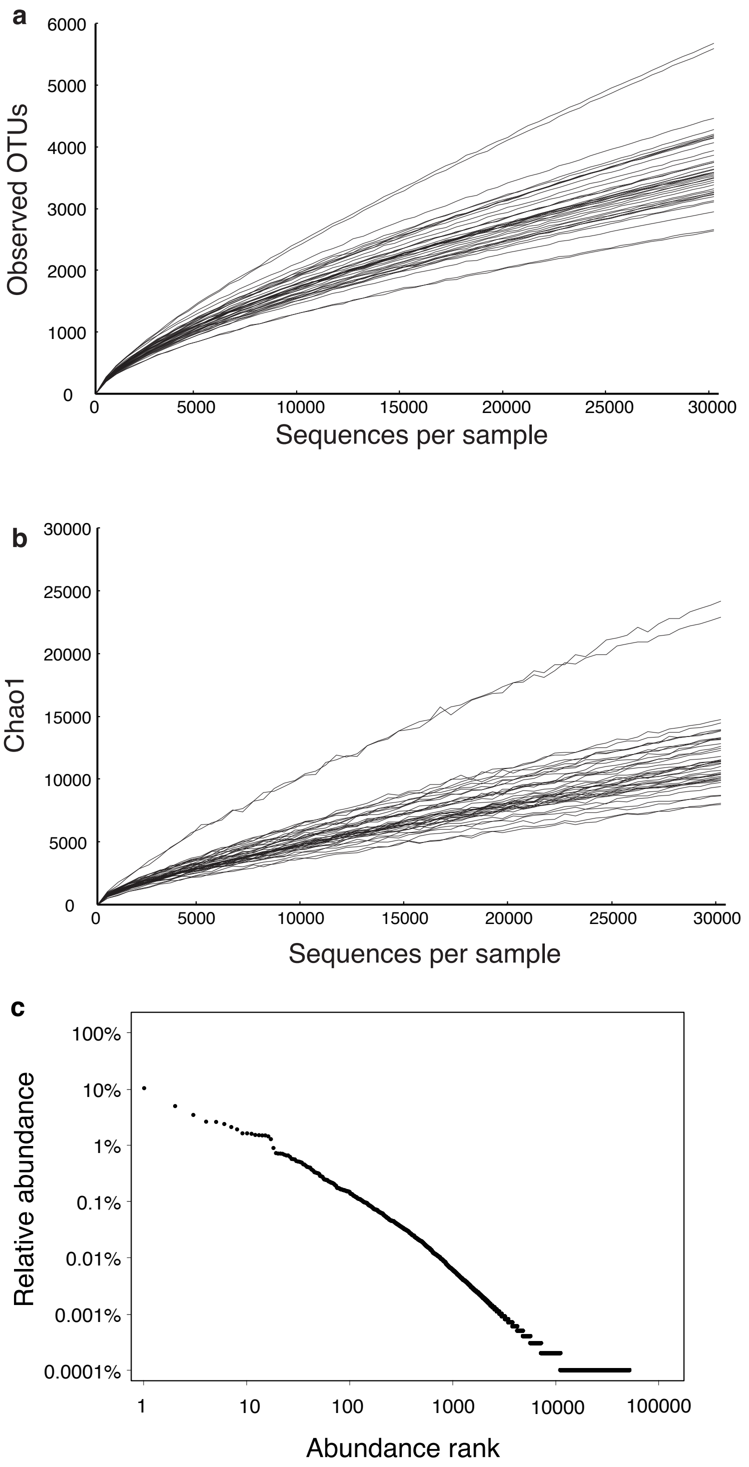

Supplementary Figure S2. Rarefaction curves of the 35 AD samples and 6 AS samples based on (**a**)observed OTUs and (**b**) Chao1 index. (**c**) Rank abundance curve of OTUs in AD.

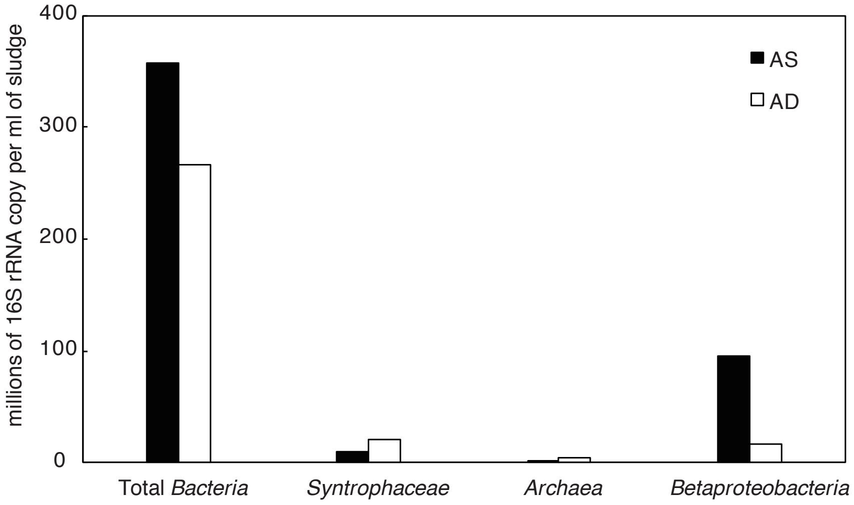

Supplementary Figure S3. Resultus of qPCR targeting total Bacteria, *Syntrophaceae* (positive μ), *Archaea* (positive μ), and *Betaproteobacteria* (negative μ).

Supplementary Table S1. Composition of feed sludge to AD

Sludge source	Flow rate (million gallons per day)	Total solids (%)	Volatile solids (%)	Percentage of volatile solids content in AD feed (%)
Activated sludge	20.03	2.83	53.10	76.10
Primary sludge	4.05	2.96	58.22	17.62
Imhoff tank sludge	0.15	6.56	46.01	5.15
Wasted sludge from another WRP	2.16	1.23	76.72	1.13

Supplementary Table S2. Sample calendar

	Monday	Tuesday	Wednesday	Thursday	Friday	Saturday	Sunday
	1[1,6,14]	2	3[1,6,14]	4	5[1,6,14,AS-A, AS-D]	6	7
	8[1]	9	10	11	12[1,AS-A, AS-D]	13	14
December, 2014	15[1,6,14]	16[1,6,14]	17	18	19[1,6,14,AS-A, AS-D]	20	21
	22[1,6,14]	23	24[1,6,14]	25	26	27	28
	29[1,6,14]	30	31	…			
January, 2015			…	29[1,6,14]			

Supplementary Table S3. Alpha-diversity estimations of AD and AS community

	No. of samples	Total No. of OTUs	Alpha-diversity estimators per sample					
			No. of OTUs	Singletons	Doubletons	Chao1	Shannon	Good's coverage
AD	35	51091	3523±68	2253±55	364±8	11145±296	8.09±0.05	0.92±0.002
AS	6	18413	4063±341	3246±334	450±25	16486±2248	8.66±0.10	0.89±0.011

Supplementary Table S4. Core populations with closely related isolates

OTU ID	Specific growth rate (d^{-1})	Related isolates	Accession No.	Similarity	Phyla	Physiological traits: Anaerobic growth	Physiological traits: Metabolic characteristic	Reference
135728	0.035	*Methanolinea mesophila* str. TNR	NR_112799.1	97%	*Euryarchaeota*	++	methanogenic	1
4062	0.014	*Methanosaeta concilii* str. GP6	NR_102903.1	99%	*Euryarchaeota*	++	methanogenic	2
136776	0.011	*Parabacteroides chartae* str. NS31-3	NR_109439.1	100%	*Bacteroidetes*	++	fermentative	3
40556	-0.051	*Niabella soli* str. NBRC106409	AB682425.1	98%	*Bacteroidetes*	-	heterotrophic	4
13323	-0.062	*Ferruginibacter yonginensis* str. HME8442	NR_133743.1	97%	*Bacteroidetes*	-	heterptrophic	5
219	0.041	*Gracilibacter thermotolerans* str. YJL-S1	NR_115693.1	97%	*Firmicutes*	++	fermentative	6
1667	-0.153	*Trichococcus pasteurii* str. KoTa2	NR_036793.1	100%	*Firmicutes*	+	fermentative	7
74151	0.040	*Holophaga foetida* str. TMBS4	NR_036891.1	99%	*Acidobacteria*	++	homoacetogenic	8
17158	-0.450	*Zoogloea caeni* str. EMB43	NR_043795.1	100%	*Proteobacteria*	+	heterotrophic	9
21907	-0.006	*Thauera phenylacetica* str. B4P	NR_027224.1	100%	*Proteobacteria*	+	heterotrophic	10
21928	-0.178	*Dechloromonas hortensis* str. ED1	NR_042090.1	99%	*Proteobacteria*	+	heterotrophic	11
57204	-0.084	*Methylophilus methylotrophus* str. HME9441	KF911346.1	99%	*Proteobacteria*	-	heterotrophic	12
109328	-0.186	*Albidiferax ferrireducens* str. TB-2	HG003356.1	100%	*Proteobacteria*	+	heterotrophic	13
21900	-0.105	*Methylibium petroleiphilum* str. HMF2787	KP099963.1	99%	*Proteobacteria*	-	heterotrophic	14
154343	-0.205	*Thermomonas carbonis* str. GZ436	NR_134219.1	100%	*Proteobacteria*	+	heterotrophic	15
137720	-0.092	*Arenimonas subflava* str. PYM3-14T	NR_135888.1	99%	*Proteobacteria*	-	heterotrophic	16
39663	-0.098	*Rhodobacter blasticus* str. ATCC33485	NR_115533.1	98%	*Proteobacteria*	+	photoorganotrophic	17
98830	-0.036	*Sphingopyxis contaminans* str. JC216	NR_134183.1	100%	*Proteobacteria*	-	heterotrophic	18
137717	0.036	*Smithella propionica* str. LYP	NR_024989.1	98%	*Proteobacteria*	++	syntrophic	19
52700	0.039	*Smithella propionica* str. LYP	NR_024989.1	98%	*Proteobacteria*	++	syntrophic	19
107171	0.039	*Syntrophorhabdus aromaticivorans* str. UI	NR_041306.1	98%	*Proteobacteria*	++	syntrophic	20

++ obligate anaerobic; + facultative anaerobic; - aerobic

Supplementary Table S5. Core populations without closely related isolates

OTU ID	Specific growth rate (d^{-1})	Closely related clone	Accession No.	Similarity	Phyla
143489	0.040	Uncultured archaeon clone DH45	KC676306.1	100%	*Euryarchaeota*
89271	0.040	Uncultured bacterium clone E103	JF428976.1	98%	*Verrucomicrobia*
171316	0.041	Uncultured bacterium clone QEEB2AE01	CU918010.1	100%	*Verrucomicrobia*
99476	0.041	Uncultured bacterium clone 21a11	EF515186.1	95%	"*Ca.* Parcubacteria"
56776	0.040	Uncultured bacterium clone OD1_CD	KT025832.1	95%	"*Ca.* Parcubacteria"
72276	0.041	Uncultured bacterium clone QEDR1AG12	CU922775.1	98%	*Thermotogae*
172417	0.041	Uncultured bacterium clone RS-S-B6	AB742078.1	100%	*Thermotogae*
93628	0.037	Uncultured bacterium clone QEDS3DE08	CU921415.1	100%	*Chloroflexi*
157968	0.036	Uncultured bacterium clone EGSB_100_4-33	KJ881331.1	100%	*Chloroflexi*
16491	0.041	Uncultured Bacteroidetes bacterium clone 13Cpro-4	AB603838.1	100%	*Bacteroidetes*
93586	0.040	Uncultured bacterium clone WRPbac61	KT167012.1	100%	*Bacteroidetes*
45943	0.040	Uncultured bacterium clone MDAF11	EU214540.1	99%	*Bacteroidetes*
88141	0.041	Uncultured bacterium clone QEEB1CE01	CU917887.1	100%	*Bacteroidetes*
172421	0.041	Uncultured Bacteroidetes bacterium clone QEDS3AA05	CU921254.1	100%	*Bacteroidetes*
103258	0.040	Uncultured bacterium clone POMEbac6	KT167021.1	100%	*Bacteroidetes*
101940	0.040	Uncultured Bacteroidetes bacterium clone 13Cpro-3	AB603834.1	100%	*Bacteroidetes*
17153	0.040	Uncultured bacterium clone WRPbac40	KT167006.1	100%	*Bacteroidetes*
81519	0.040	Uncultured bacterium clone PISD-AIA06	AM982594.1	95%	*Bacteroidetes*
94033	0.038	Uncultured Bacteroidetes bacterium clone QEEB2BG02	CU918225.1	99%	*Bacteroidetes*
78197	0.037	Uncultured bacterium clone MT-95	KP663407.1	98%	*Bacteroidetes*
20287	0.036	Uncultured Bacteroidetes bacterium clone QEDR1CC06	CU922387.1	100%	*Bacteroidetes*
54922	0.035	Uncultured Bacteroidetes bacterium clone RSg13-32	AB603818.1	99%	*Bacteroidetes*
71234	0.039	Uncultured Bacteroidetes bacterium clone B58	AB780945.1	100%	*Bacteroidetes*
106400	-0.171	Uncultured bacterium clone BJ 2-78	KC551764.1	100%	*Bacteroidetes*
125932	-0.041	Uncultured bacterium clone ambient_uncontrolled-22	GU454883.1	100%	*Bacteroidetes*
143488	-0.022	Uncultured bacterium isolate DGGE gel band RB1-30	KT835500.1	100%	*Bacteroidetes*
70567	-0.132	Uncultured bacterium clone A79	HG917493.1	100%	*Bacteroidetes*
117056	0.040	Cloacimonetes bacterium JGI 0000059-L07	KJ535434.1	100%	"*Ca.* Cloacimonetes"
120628	0.041	Uncultured WWE1 bacterium clone QEDS3CA02	CU921669.1	100%	"*Ca.* Cloacimonetes"
175193	0.041	Uncultured bacterium clone QEEB2AG12	CU918398.1	100%	"*Ca.* Cloacimonetes"
103263	0.041	Uncultured Firmicutes bacterium clone QEDQ2BC11	CU923016.1	100%	*Firmicutes*

3703	0.041	Uncultured bacterium clone AHPD_Bac1_C01	KJ206759.1	100%	*Firmicutes*
117055	0.040	Uncultured Firmicutes bacterium clone B67	AB780954.1	100%	*Firmicutes*
22996	0.041	Uncultured Firmicutes bacterium clone QEDN4AE02	CU926871.1	100%	*Firmicutes*
23639	0.040	Uncultured bacterium clone ambient_uncontrolled-56	GU454917.1	100%	*Firmicutes*
58521	0.041	Uncultured bacterium clone BSA2B-10	AB175382.1	99%	*Firmicutes*
151392	0.041	Uncultured bacterium clone NBBME0308_94	JQ072357.1	100%	*Tenericutes*
99809	0.041	Uncultured bacterium clone QEEB3CH05	CU917592.1	99%	*Tenericutes*
118600	0.040	Uncultured bacterium clone QEDR2CG01	CU922788.1	100%	*Tenericutes*
84581	0.040	Uncultured bacterium clone BP5-7	KF564577.1	100%	MVP-15
179065	0.030	Uncultured Spirochaetes bacterium clone QEDN2BE08	CU925939.1	100%	*Spirochaetes*
100217	0.041	Uncultured bacterium clone OTU-BMAR60-6	KF493717.1	100%	*Spirochaetes*
120133	0.040	Uncultured bacterium clone b79	KJ578066.1	94%	*Spirochaetes*
128063	0.040	Uncultured bacterium clone MCB-TH-17	KP226617.1	100%	*Spirochaetes*
137220	0.041	Bacterium enrichment culture clone EtOH-82	FJ799137.1	99%	*Spirochaetes*
31037	0.040	Uncultured Spirochaetes bacterium clone QEDS2AC07	CU921278.1	99%	*Spirochaetes*
175425	0.040	Uncultured Spirochaetes bacterium clone QEDN7DB02	CU926998.1	99%	*Spirochaetes*
143491	0.039	Uncultured Proteobacteria bacterium clone QEDP3DE04	CU924126.1	100%	*Proteobacteria*
134233	-0.078	Uncultured bacterium clone CN-13	KP054226.1	100%	*Proteobacteria*
157865	-0.229	Uncultured bacterium clone D87	KJ808191.1	100%	*Proteobacteria*
137722	-0.035	Uncultured bacterium clone Rap1_6A	EF192877.1	100%	*Proteobacteria*
114426	-0.165	Uncultured Proteobacterium clone D7	KC633531.1	100%	*Proteobacteria*
162138	-0.052	Uncultured bacterium clone IAN41	KF428095.1	100%	*Proteobacteria*

Supplementary Table S6. Primers and reactions used for qPCR targeting 16S rRNA gene

Target group	Primer	Sequence	*E. coli* position	qPCR amplification condition	Reference
Bacteria	341F	CCTACGGGAGGCAGCAG	341-357	95°C for 15 s, 60°C for 30 s, 40 cycles	[21]
	518R	ATTACCGCGGCTGCTGG	518-534		
Syntrophaceae	Syn424F	ATCGTAAAGCTCTGTCGG	424-441	95°C for 15 s, 57°C for 15 s, 72°C for 40 s, 40 cycles	[22]
	Syn827R	CYCYCAACACCTAGTGAA	822-839		
Archaea	Arc967F	AATTGGCGGGGGAGCAC	967-983	95°C for 10 s, 60°C for 15 s, 40 cycles	[23]
	Arc1060R	GGCCATGCACCWCCTCTC	1060-1077		
Betaproteobacteria	Eub338F	ACTCCTACGGGAGGCAGCAG	338-357	95°C for 15 s, 55°C for 30 s, 72°C for 30 s, 40 cycles	[24]
	Bet680R	TCACTGCTACACGYG	680-694		

Reference:

1 Sakai, S. *et al.* Methanolinea mesophila sp. nov., a hydrogenotrophic methanogen isolated from rice field soil, and proposal of the archaeal family Methanoregulaceae fam. nov. within the order Methanomicrobiales. *Int J Syst Evol Micr* **62**, 1389-1395 (2012).

2 Patel, G. B. & Sprott, G. D. Methanosaeta concilii gen. nov., sp. nov.("Methanothrix concilii") and Methanosaeta thermoacetophila nom. rev., comb. nov. *International journal of systematic bacteriology* **40**, 79-82 (1990).

3 Tan, H.-Q. *et al.* Parabacteroides chartae sp. nov., an obligately anaerobic species from wastewater of a paper mill. *Int J Syst Evol Micr* **62**, 2613-2617 (2012).

4 Weon, H.-Y. *et al.* Niabella soli sp. nov., isolated from soil from Jeju Island, Korea. *Int J Syst Evol Micr* **58**, 467-469 (2008).

5 Lee, B.-I., Kang, H., Kim, H., Joung, Y. & Joh, K. Ferruginibacter yonginensis sp. nov., isolated from a mesotrophic artificial lake. *Int J Syst Evol Micr* **64**, 846-850 (2014).

6 Lee, Y.-J. *et al.* Gracilibacter thermotolerans gen. nov., sp. nov., an anaerobic, thermotolerant bacterium from a constructed wetland receiving acid sulfate water. *Int J Syst Evol Micr* **56**, 2089-2093 (2006).

7 Liu, J.-R. *et al.* Emended description of the genus Trichococcus, description of Trichococcus collinsii sp. nov., and reclassification of Lactosphaera pasteurii as Trichococcus pasteurii comb. nov. and of Ruminococcus palustris as Trichococcus palustris comb. nov. in the low-G+ C gram-positive bacteria. *Int J Syst Evol Micr* **52**, 1113-1126 (2002).

8 Liesack, W., Bak, F., Kreft, J.-U. & Stackebrandt, E. Holophaga foetida gen. nov., sp. nov., a new, homoacetogenic bacterium degrading methoxylated aromatic compounds. *Archives of Microbiology* **162**, 85-90 (1994).

9 Shao, Y. *et al.* Zoogloea caeni sp. nov., a floc-forming bacterium isolated from activated sludge. *Int J Syst Evol Micr* **59**, 526-530 (2009).

10 Mechichi, T., Stackebrandt, E., Gad'on, N. & Fuchs, G. Phylogenetic and metabolic diversity of bacteria degrading aromatic compounds under denitrifying conditions, and description of Thauera phenylacetica sp. nov., Thauera aminoaromatica sp. nov., and Azoarcus buckelii sp. nov. *Archives of microbiology* **178**, 26-35 (2002).

11 Wolterink, A. *et al.* Dechloromonas hortensis sp. nov. and strain ASK-1, two novel (per) chlorate-reducing bacteria, and taxonomic description of strain GR-1. *Int J Syst Evol Micr* **55**, 2063-2068 (2005).

12 Kalyuzhnaya, M. G. *et al.* Novel methylotrophic isolates from lake sediment, description of Methylotenera versatilis sp. nov. and emended description of the genus Methylotenera. *Int J Syst Evol Micr* **62**, 106-111 (2012).

13 Finneran, K. T., Johnsen, C. V. & Lovley, D. R. Rhodoferax ferrireducens sp. nov., a psychrotolerant, facultatively anaerobic bacterium that oxidizes acetate with the reduction of Fe (III). *Int J Syst Evol Micr* **53**, 669-673 (2003).

14 Nakatsu, C. H. *et al.* Methylibium petroleiphilum gen. nov., sp. nov., a novel methyl tert-butyl ether-degrading methylotroph of the Betaproteobacteria. *Int J Syst Evol Micr* **56**, 983-989 (2006).

15 Wang, L., Zheng, S., Wang, D., Wang, L. & Wang, G. Thermomonas carbonis sp. nov., isolated from the soil of a coal mine. *Int J Syst Evol Micr* **64**, 3631-3635 (2014).

16 Makk, J. *et al.* Arenimonas subflava sp. nov., isolated from a drinking water network, and emended description of the genus Arenimonas. *Int J Syst Evol Micr* **65**, 1915-1921 (2015).
17 Eckersley, K. & Dow, C. S. Rhodopseudomonas blastica sp. nov.: a member of the Rhodospirillaceae. *Journal of General Microbiology* **119**, 465-473 (1980).
18 Subhash, Y., Sasikala, C. & Ramana, C. V. Sphingopyxis contaminans sp. nov., isolated from a contaminated Petri dish. *Int J Syst Evol Micr* **64**, 2238-2243 (2014).
19 Liu, Y., Balkwill, D. L., Aldrich, H. C., Drake, G. R. & Boone, D. R. Characterization of the anaerobic propionate-degrading syntrophs Smithella propionica gen. nov., sp. nov. and Syntrophobacter wolinii. *International journal of systematic bacteriology* **49**, 545-556 (1999).
20 Qiu, Y.-L. *et al.* Syntrophorhabdus aromaticivorans gen. nov., sp. nov., the first cultured anaerobe capable of degrading phenol to acetate in obligate syntrophic associations with a hydrogenotrophic methanogen. *Appl Environ Microb* **74**, 2051-2058 (2008).
21 Sonthiphand, P. & Neufeld, J. D. Evaluating primers for profiling anaerobic ammonia oxidizing bacteria within freshwater environments. *PloS one* **8**, e57242 (2013).
22 Gray, N. *et al.* The quantitative significance of Syntrophaceae and syntrophic partnerships in methanogenic degradation of crude oil alkanes. *Environ Microbiol* **13**, 2957-2975 (2011).
23 Bayer, K., Kamke, J. & Hentschel, U. Quantification of bacterial and archaeal symbionts in high and low microbial abundance sponges using real-time PCR. *FEMS microbiology ecology* **89**, 679-690 (2014).
24 Philippot, L., Tscherko, D., Bru, D. & Kandeler, E. Distribution of high bacterial taxa across the chronosequence of two alpine glacier forelands. *Microbial ecology* **61**, 303-312 (2011).
